# Vienna Talking Faces (ViTaFa): A multimodal person database with synchronized videos, images, and voices

**DOI:** 10.3758/s13428-023-02264-5

**Published:** 2023-11-10

**Authors:** Christina Krumpholz, Cliodhna Quigley, Leonida Fusani, Helmut Leder

**Affiliations:** 1 Department of Cognition, Emotion, and Methods in Psychology, Faculty of Psychology, University of Vienna, Liebiggasse 5, 1010 Vienna, Austria; 2 Konrad Lorenz Institute of Ethology, University of Veterinary Medicine, Vienna, Austria; 3 Department of Behavioural and Cognitive Biology, University of Vienna, Vienna, Austria; 4 Vienna Cognitive Science Hub, University of Vienna, Vienna, Austria

**Keywords:** Face, Voice, Audiovisual integration, Social perception, Attractiveness

## Abstract

Social perception relies on different sensory channels, including vision and audition, which are specifically important for judgements of appearance. Therefore, to understand multimodal integration in person perception, it is important to study both face and voice in a synchronized form. We introduce the Vienna Talking Faces (ViTaFa) database, a high-quality audiovisual database focused on multimodal research of social perception. ViTaFa includes different stimulus modalities: audiovisual dynamic, visual dynamic, visual static, and auditory dynamic. Stimuli were recorded and edited under highly standardized conditions and were collected from 40 real individuals, and the sample matches typical student samples in psychological research (young individuals aged 18 to 45). Stimuli include sequences of various types of spoken content from each person, including German sentences, words, reading passages, vowels, and language-unrelated pseudo-words. Recordings were made with different emotional expressions (neutral, happy, angry, sad, and flirtatious). ViTaFa is freely accessible for academic non-profit research after signing a confidentiality agreement form via https://osf.io/9jtzx/ and stands out from other databases due to its multimodal format, high quality, and comprehensive quantification of stimulus features and human judgements related to attractiveness. Additionally, over 200 human raters validated emotion expression of the stimuli. In summary, ViTaFa provides a valuable resource for investigating audiovisual signals of social perception.

Real-life encounters with other individuals occur in various sensory modalities and involve dynamic changes in sensory signals over time. Our interactions extend far beyond mere facial expressions, as we engage with voice, body, and scent in a dynamic perception of our counterparts that drives our impressions. In order to empirically study the integration of different types of person appearances, we require stimulus material that is multimodal and dynamic. An overview of existing stimulus material will show that existing stimulus material is relatively scarce in terms of publicly available resources that combine multiple sensory modalities and offer realistic dynamic stimuli. This might have limited the study of interactions between and integration of sensory channels. Our new database provides synchronized dynamic audiovisual material that enables the investigation of two sensory channels by incorporating dynamic information from voice and face.

Despite acknowledging the necessity for a more comprehensive understanding of the complex signaling of attractiveness, previous studies have predominantly focused on examining single modalities in isolation, with a particular emphasis on face (Little, 2014), voice (Hill & Puts, 2016; Pisanski & Feinberg, 2018), or scent (Ferdenzi et al., 2020). While these studies provide valuable insights into the contributions of specific sensory modalities to attractiveness judgements, they are limited in their ability to capture the complexity of real-life encounters, which involve the simultaneous processing of several sensory modalities. Studies using visual and auditory stimulus material (e.g., Mook & Mitchel, 2019; Raines et al., 1990) suggest that the perception of attractiveness relies on the interplay of multiple modalities, motivating the need for multisensory stimulus material.

Even more surprising than the lack of attention given to multisensory processing of attractiveness is that many studies rely on static stimuli, even though previous work has highlighted issues with external validity of findings with static stimuli to real-life situations (Garrido et al., 2017; Horstmann & Ansorge, 2009). We perceive others in motion, with continuously changing input about temporal aspects of facial movement (Fujimura & Suzuki, 2010) and intermodal cues such as lip movement (Sumby & Pollack, 1954), rate (Munhall et al., 1996), and rhythm (Bahrick & Lickliter, 2004), which are all fundamental components of nonverbal communication. However, these features are all absent in static images or static images paired with voice recordings (Lander, 2008). Moreover, previous work has highlighted differences in identifying emotional expressions between dynamic and static face presentations, underscoring the importance of motion as a crucial factor in how humans perceive other people (Fiorentini & Viviani, 2011; Horstmann & Ansorge, 2009). Collectively, these studies suggest that previous findings in person perception based on static stimulus material might not translate to real-world scenarios, urging the use of dynamic stimuli in future studies.

We developed a new database to allow for more ecologically valid research on multimodal attractiveness and person perception in general. Creating such stimulus material can be costly and time-consuming, and existing databases are often unsuitable for studying multimodal processing of attractiveness because they only contain unimodal and/or static material and target other research fields, such as person or emotion recognition. Our database provides face and voice material of 40 actors in various expressions (neutral, happy, angry, sad, flirtatious) and content (vowels, words, sentences), available in multiple formats: unimodal in the form of static images, voice recordings, and muted dynamic videos; and multimodal in the form of dynamic, synchronized audiovisual videos. Below, an overview of available databases for face and voice research and their limitations highlights the importance of our new database in advancing the study of multisensory processing of attractiveness.

## Available face and voice databases

Face image databases are used to study face processing, recognition, identification, emotion recognition, memorability, social perception, and attractiveness in human participants and for the development of automated computational solutions. Different research areas require stimulus material with specific properties, which are reflected in the design and composition of available databases. An overview of all the databases reviewed in the following sections can be found in the data repository of this paper (https://osf.io/6rdb3).

Databases developed for the investigation of facial or person recognition often contain multiple stimuli of the same person recorded under various conditions to acknowledge variation in how faces appear (Bruce, 1994). Images of people have been recorded at several different time points, e.g., in the AT&T database (Samaria & Harter, 1994), the CMU Multi-PIE database (Gross et al., 2008), or the FERET database (Phillips et al., 1998). Varying lighting conditions have been employed, e.g., in the CAS-PEAL face database (Gao et al., 2008), and target samples recorded from different viewpoints, e.g., in the CMU Multi-PIE database (Gross et al., 2008) or in the Face Place(s) database (Righi et al., 2012). Photographs of different facial expressions have been provided, e.g., in the Face Place(s) database (Righi et al., 2012), the FG-NET network (Wallhoff et al., 2006), and the Meissner African American and Caucasian Male Sets (Meissner et al., 2005), with different facial details, accessories, or disguises, e.g., in the AT&T database (Samaria & Harter, 1994), the CAS-PEAL database (Gao et al., 2008), and the Face Place(s) database (Righi et al., 2012), or with varying poses, e.g., in the CAS-PEAL database (Gao et al., 2008). Some of these large-scale databases have repurposed pre-existing material such as photographs of celebrities (FaceScrub database by Ng & Winkler, 2014), algorithm-derived sets of images of the same person retrieved from the Internet (Labeled Faces in the Wild by Huang et al., 2008), or even millions of hours of utterances retrieved from interview uploads to YouTube (VoxCeleb database by Nagrani et al., 2020) to investigate speaker recognition under noisy and unconstrained conditions.

Databases created for the purpose of emotion recognition research mainly contain facial images expressing the six basic emotions – angry, disgusted, fearful, happy, sad, surprised – and a neutral condition, whereby the emotion could either be spontaneous, as for instance in the FACES database (Ebner et al., 2010), or posed as in the Child Affective Facial Expression Set (LoBue & Thrasher, 2015), Karolinska Directed Emotional Faces database (Calvo & Lundqvist, 2008), Montreal Set of Facial Displays of Emotion (here, surprised is swapped with embarrassed; Beaupré et al., 2000), NimStim Set of Facial Expressions (including a calm condition; Tottenham et al., 2009), Radboud Faces Database (Langner et al., 2010), or in the Yonsei Face Database (Chung et al., 2019). The Complex Emotion Expression Database (Benda & Scherf, 2020) additionally includes images of complex emotions such as flirtatious, attracted, or desirous, allowing the investigation of emotions related to more nuanced social behavior and inner thoughts. Apart from these databases relying on static images, more recent publications offer dynamic muted video material of emotional expressions, e.g., Amsterdam Dynamic Facial Expression Set (van der Schalk et al., 2011), Belfast Natural Induced Emotion Dataset (Sneddon et al., 2012), Dynamic FACES dataset (artificial videos generated from images; Holland et al., 2019), Faces and Motion Exeter Database (Longmore & Tree, 2013), Ryerson Audio-Visual database of Emotional Speech and Song (Livingstone & Russo, 2018), or the SAVE database (Garrido et al., 2017).

However, few databases contain dynamic audiovisual material of emotional expressions, i.e., videos simultaneously containing visual information from the face and auditory information from the voice. The MMI Facial Expression Database (Pantic et al., 2005) contains video clips of spontaneous (and therefore unstandardized) emotional reactions of 75 subjects to a given stimulus, whereas the SAVEE Database (Haq & Jackson, 2010) contains video clips of four subjects reading out 15 different sentences in seven different posed expressions.

Other databases have been developed to study social perception in a broader framework including attractiveness, whereby most rely on unimodal stimuli. Most of these databases are characterized by the fact that they include extensive validation data on the perception of the stimulus material (cf. Chicago Face Database by Ma et al., 2015; Bogazici Face Database by Saribay et al., 2018; Face Research Lab – London Set by DeBruine & Jones, 2021; SAVE database by Garrido et al., 2017; Geneva Faces and Voices by Ferdenzi et al., 2015). These validation data include subjective ratings on dimensions such as attractiveness, trustworthiness, femininity, health, and assessments of age, gender, or ethnicity. They further include objective measures such as facial landmarks, face measurements, symmetry, or averageness. Different ethnicities and their perceptions also matter when it comes to social perception (Lord et al., 2019; McKone et al., 2021), which is why some databases specifically include people from different ethnic backgrounds, such as the American Multiracial Face Database (Chen et al., 2021), Japanese and Caucasian Facial Expression of Emotion and Neutral Faces databases (Matsumoto & Ekman, 1994), or the MR2 Face Database (Strohminger et al., 2016). Recently, computer-generated databases have become increasingly important because they allow precise manipulation, for example of individual facial features, with a high degree of standardization. The AI Generate Faces database (Karras et al., 2018) was created using generative adversarial networks, whereby images can be manipulated using a wide range of dimensions. The Todorov Synthetic Faces Databases (e.g. validated in Todorov et al., 2013) contain computer-generated images manipulated in face shape, reflectance, ethnicity, or different trait dimensions in various degrees. Software has also been developed that can be applied to existing databases in order to manipulate 2D or 3D face models to change identity, pose, and expression and to create caricatures, average face models, or standardized stimulus sets (Face Research Toolkit by Hays et al., 2020; Psychomorph, Tiddeman, 2011). While offering relevant validation data for attractiveness research, most of these social perception databases rely on static images. For voices, there are very few available databases which contain voice recordings of different speakers. These include the Jena Speaker Set (Zäske et al., 2020), the Montreal Affective Voices database (Belin et al., 2008), the Oxford Vocal Sounds Database (Parsons et al., 2014), and the UCL Speaker Database (Markham & Hazan, 2002), which all allow investigation of voice perception in social contexts.

This survey of existing databases shows that the focus is on unimodal stimulus presentation, mainly still images of faces, although in real-world social encounters, we rarely, if ever, encounter only static facial images. Notable exceptions are the Geneva Faces and Voices database (Ferdenzi et al., 2015) and the VidTIMIT Audio-Video Dataset (Sanderson & Lovell, 2009), which offer multimodal material in the form of muted videos and voice recordings, and the SAVE database (Garrido et al., 2017) which contains dynamic muted videos and still images. Interestingly, their comparison between both stimulus types (still images and dynamic muted videos) revealed significant differences regarding assessments of attractiveness, familiarity, genuineness, and intensity. Further studies showed facilitation effects in affective processing for dynamic facial stimuli compared to still images (Cunningham & Wallraven, 2009; Rubenstein, 2005; Wehrle et al., 2000). These results suggest that still images and muted videos are processed differently, possibly due to variations in evaluative criteria or the relative saliency of specific features. This emphasizes the need for dynamic stimulus material in social perception research. Since real-life expressions involve action, they are most likely to be processed like dynamic material rather than static material (van der Schalk et al., 2011). Audiovisual databases are rare and mainly developed for other research purposes such as speech perception (GRID audiovisual sentence corpus by Cooke et al., 2006), deception detection (Miami University Deception Detection Database; Lloyd et al., 2019), or emotion recognition (SAVEE database with videos over a longer time period of only four subjects, Haq & Jackson, 2010; RAVDESS database, Livingstone & Russo, 2018).

In this context, our goal was to create and provide a new high-quality audiovisual database, the Vienna Talking Faces (ViTaFa) database, with a special focus on the study of multimodal signals of attractiveness but also applicable to a broader field of face and voice processing research. The ViTaFa database meets the following criteria:

- Original stimuli were collected from real individuals, including various types of stimuli from the same individual.
- Different stimulus modalities were included: unimodal visual static and dynamic; unimodal auditory; multimodal audiovisual dynamic.
- Various content was recorded, including German sentences, words, reading passages, vowels, and language-unrelated pseudo-words.
- Content was recorded with different emotional expressions – neutral, happy, angry, sad – and flirtatious expression.
- Stimuli originate from a sample that matches most student samples in psychological research: young females and males aged 18 to 45.
- All stimuli were collected under highly standardized recording and editing conditions.
- The database is freely accessible for academic non-profit research.

Among publicly available databases, ViTaFa is therefore characterized by its multimodal format, high quality, and usefulness for research into social communication including attractiveness. Our selection of emotional expressions was purposefully designed to create a versatile multimodal database, tailored to address various aspects of research into person perception. Extensive prior research has underscored the significant impact of facial and vocal cues to emotional expressions on person perception. Notably, happy facial expressions have consistently been associated with a multitude of positive social attributions, encompassing qualities such as sincerity, competence, sociability (Reis et al., 1990), trustworthiness (Calvo et al., 2018), familiarity, and overall positivity (Garrido et al., 2017). This influence extends to attractiveness perception, where studies have consistently shown that individuals tend to rate happy facial expressions as more attractive than neutral or angry expressions (Calvo et al., 2018; Garrido et al., 2017; Ho & Newell, 2020; Kaisler et al., 2020; Lindeberg et al., 2019; Reis et al., 1990; Ueda et al., 2016). Moreover, these investigations suggest that attractiveness perception is intricately linked to emotional expression, with the valence (positive or negative) and intensity of the emotion further shaping these judgments (Ueda et al., 2016). This motivated our decision to incorporate not only a neutral emotional condition but also conditions with positive valence (happy) and negative valence (sad, angry). In recognition of the importance of flirting as a pivotal behavior in mate choice, intimately linked to mating success and the development of relationships (Apostolou, 2021; Apostolou et al., 2019), we have included an additional flirtatious condition. This addition underscores our dedication to furnishing a comprehensive resource for investigating attractiveness and for broader research into person perception in various social and interpersonal contexts.

Beyond visual/auditory material, it contains comprehensive quantification of standard stimulus features and human judgements of dimensions of social perception related to attractiveness research, as well as validation of emotion expression and recognizability by over 200 human raters. Below we describe how we collected and processed the stimuli, and the process of creating subjective evaluations and objective measurements of the stimulus material.

## Materials and methods

### Actors

We included material from 20 women and 20 men in the database. Individuals were recruited through Facebook advertisements, acting schools, and the researchers’ circle of acquaintances. During the recruitment process we targeted amateur actors as well as people we believed would reliably be able to produce credible stimulus material. We required actors to be fluent German speakers between the age of 18 and 45 (*M* = 28.55 years, *SD* = 6.40), to identify as male or female, and to be heterosexual and without any facial deformities such as plasters or wounds. Heterosexuality was required, as we included a flirting condition in the ViTaFa database. Literature on different sexual orientations revealed differences in sociosexual behavior across orientation (Schmitt, 2013), which could confound behavior in the flirting condition (Back et al., 2011). We excluded psychology students from the University of Vienna to avoid that they might be recognized in future studies with student participants. All actors either wore their own black shirts or were provided with a black shirt by the researcher and were asked to wear no make-up or simple make-up. To keep natural variance, we allowed visual ornaments. Nine actors wore earrings, one wore a visible hairclip, two had piercings, and 16 had a beard. Four wore simple make-up. Actors received financial compensation of 10€ per hour. Of 47 people that were invited to the experiment, we had to exclude seven: one requested exclusion of her videos, two were accidentally recorded with a wrong frame rate, one was too tall to fit the camera set-up, two showed insufficient acting skills, and one because the camera system crashed. Two actors were recorded twice because of wrong camera settings in the first session such that their second recordings were included in the database.

A printed information sheet notified actors that their faces, voices, and the upper parts of their bodies were going to be recorded, that this material could be used for future studies, and that this material would be saved in a database accessible to other researchers upon request and for academic research only. We emphasized that future use requires researchers to sign a confidentiality agreement and accept terms of use (available via https://osf.io/bmsye) in which they commit to only use the database for scientific purposes, to not distribute it to other researchers, and to not depict any material in publications except provided sample material. Actors were also informed that the material could be manipulated in the future for research purposes. We also informed them that they can withdraw their consent to be included in the database at any point, which would, however, occur only for future downloads of the database after their stimulus material is withdrawn, as we cannot guarantee that other researchers have not already downloaded and used the material in previous studies. All actors gave written informed consent.

### Development of the stimulus set

#### Apparatus

**Fig. 1:**
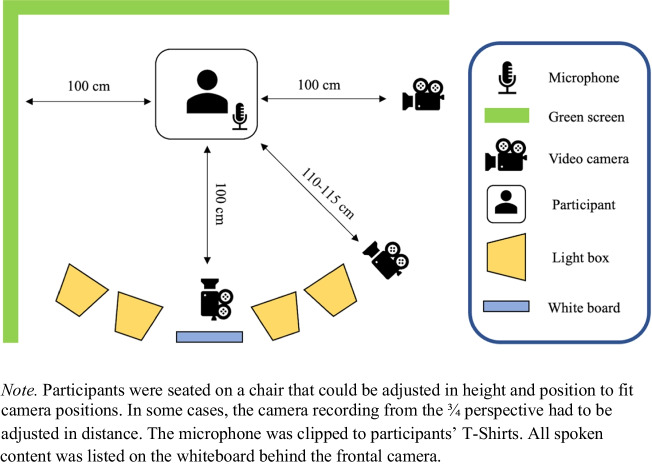
Filming setup

For stimulus collection, we employed the setup visualized in Fig. 1. Three cameras (Basler acA1920-155uc) were mounted on tripods so that the camera lenses were centered on the average height of the actors in a seated position (110 cm). The cameras were positioned to record from a frontal, a profile, and a ¾ perspective with an approximate distance to the actor of 100 cm for the frontal and profile perspectives (25-mm camera lenses) and 110–115 cm for the ¾-perspective (35-mm camera lens). Profile and ¾ perspective were always recorded from the same side, capturing mainly the left half of the face. The video frame rate was set to 30 frames per second, exposure to 0.02 s. Audio was recorded using Sennheiser SK 100 G3 pocket transmitter microphones and a Zoom H5 Handy recorder with a sampling rate of 48 kHz and quantization of 16 bits. The volume was set individually for each actor and normalized later in post-processing. The cameras and audio recorder were connected to a Motif Video Recording System (Loopbio GmbH, Vienna, Austria) for synchronized recording, which was controlled remotely via a wireless connection (for a more detailed description of the recording system see Janisch et al., 2021). Four lighting soft boxes (set at 5500 Kelvin to simulate daylight) were installed facing the actors (see Fig. 1). A green screen was placed behind the actors to have a consistent background in all camera angles. The actor’s chair was height adjustable, and its position could be adjusted to the front or back depending on the height of the actors; sometimes, it was necessary to adjust the camera positions. Behind the frontal camera, we placed a whiteboard with the instructions clearly readable. During the entire recording, the experimenter sat behind this whiteboard, monitored the camera settings using the recording software, and gave instructions.

#### Procedure

After the potential actors registered interest in participation, we sent them information material about the database and their tasks during the recordings. All actors agreed to participate and were invited to the Faculty of Psychology at the University of Vienna, where the filming environment was set up. Several days before the appointment, the actors received an exact script containing the spoken content and emotional conditions under which they would be filmed to familiarize them with the procedure. They were instructed to prepare and to rehearse the script at home if needed. Upon arrival, the actors were asked to read through the consent form and to fill out a demographic questionnaire (results are available via https://osf.io/87n3x). We assessed their current age, prior acting experience, relationship status, and, for females, current stage of their menstrual cycle. The inclusion of the latter two variables was motivated by ViTaFa's intended use in attractiveness research, as they have previously been demonstrated to influence attractiveness perception (e.g., O’Hagen et al., 2003; Puts et al., 2012). We also asked how often actors had rehearsed the script. Twenty actors reported one to two training sessions, 13 actors reported no training sessions, and seven actors reported three or more training sessions. The experimenter additionally orally informed them about the aim of the study and usage rights of the produced material to avoid any misunderstandings before the consent form was signed. The actors were then asked to sit on the chair in the filming setup in a comfortable position. Doors and windows were closed and all light sources except the light boxes were shut down. Chair height and distance to the green screen, and if necessary camera height and distance, were adjusted to reach a similar video composition for all actors. In video post-processing, this was further adjusted through video cropping. The actors were then instructed on the procedure, which was identical for all recording sessions. We recorded the same order of emotion conditions with short breaks in between: neutral, happy, sad, angry, and flirtatious. During the entire filming process, the actors were given the opportunity to take breaks whenever they needed. We used semi-standardized instructions for each emotional condition offering example situations that actors should imagine while performing.

**Fig. 2:**
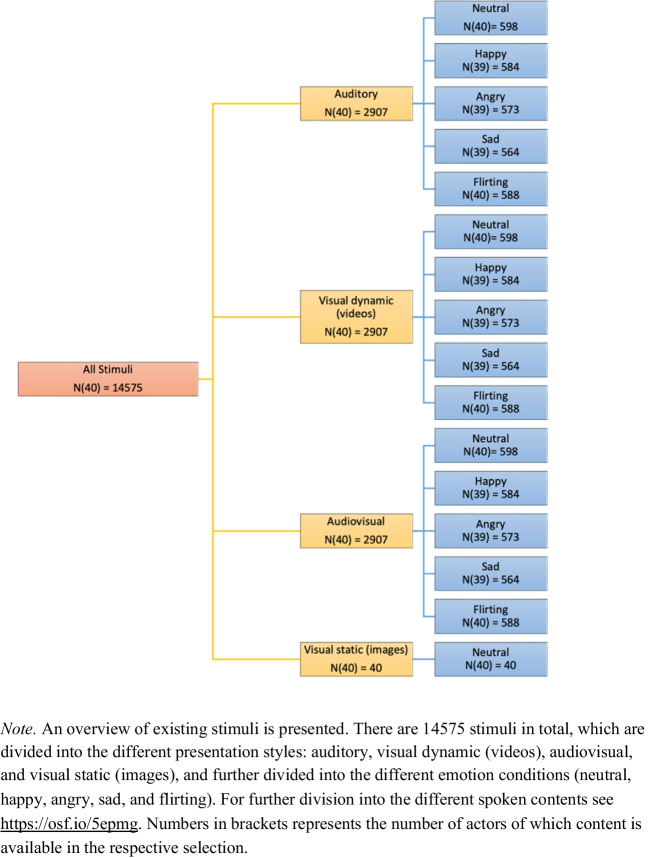
Overview of available stimuli

Within each emotion condition, actors were asked to perform a variety of tasks. Therefore, each task was performed in all five emotion conditions. The first task involved reciting two commonly used German phrases, *Hallo, ich bin’s* [Hello, it’s me] and *Wie geht’s dir?* [How are you?]. This was followed by two neutral sentences, *Morgens ist auf den Straßen viel los* [The streets are busy in the morning] and *Die Leute sitzen vor der Tür* [People sit outside the door]. Two words from these sentences, *Straße* [street] and *Tür* [door], were repeated by the actors after the sentences. Next, the actors were asked to recite two language-unrelated words, *bido* and *gali*. These words are phonetically and orthographically correct but have no existing meaning in German language. *Bido* has no widely recognized meaning in any other major language, and *Gali* only in a few languages (Hindi/Gujarati/Lithuanian) making them suitable for studies across cultures. Actors were then instructed to recite the vowels *a* [aː], *e* [eː], *i* [iː], *o* [oː], and *u* [uː]. To include a sentence where the actor shows interest in the possible receiver, they were instructed to recite the phrase *Willst du mit mir einen Kaffee trinken gehen?* [Do you want to go for a coffee with me?]. Finally, the actors were instructed to read the first three sentences from the German version of the fairy tale Snow White (Grimm & Grimm, 1812/1815) displayed on the whiteboard behind the camera. Every content was repeated a minimum of three times until the researcher was convinced that at least one of the recorded takes would be suitable for the database. The decision was made based on factors such as grammatical accuracy, the ability to recognize the conveyed emotion, as well as the actor’s eye gaze and body movement. Once filming was completed, the actors were thanked and provided with their financial compensation. Overall, we captured each person performing five sentences, four words, and five vowels, as well as reading a passage from Snow White, from three different camera angles and under five different emotion conditions each, resulting in a total of 9000 short videos clips. However, at present, the database contains only the 3000 videos captured from the frontal perspective, but uncut videos from the other two angles can be made available. Moreover, of the clips from the frontal perspective, 93 recordings were affected by technical issues, as the recording crashed or files were broken, which was noticed after filming and could not be repeated. Therefore, 2907 audiovisual video files are currently available. An overview of all available data files is visualized in Fig. 2 and can be found here (for optimal readability download the file and open with suitable software for .xlsx files): https://osf.io/5epmg.

#### Post-processing

Instead of recording each piece of content individually, we recorded a single video and audio file respectively per emotion and post-processed it as follows to end up with the short audiovisual videos (.mp4 files), video-only videos (.mp4 files), audio tracks (.wav files), and, for the neutral condition, static images (.png files). Original audio files contained the audio track and a synchronization signal track from the Motif camera system hardware synchronizer (one pulse per frame), which allowed cutting of corresponding portions of the full audio track and video recording. Background noise of the audio track was repeatedly reduced by 5 dB using Audacity’s (2016) noise reduction to remove a thumping sound originating from the synchronization signal. However, care was taken to preserve the natural sound of each voice by removing only those sounds that do not naturally occur in it. Using FFmpeg (The FFmpeg Developers, 2020), a loudnorm filter was applied to normalize audio clips according to the EBU R128 standard, a loudness measurement based on the integrated loudness calculated over the entire duration of the audio recording (European Broadcasting Union, 2020). Afterwards, audio tracks were synchronized with the video files with an accuracy of ± 1 video frame (33.33 ms). Subsequently, all video files were converted to .mp4 format, and each onset and offset frame of each spoken content was manually marked using the behavioural scoring function in the software Loopy (Loopbio GmbH, Vienna, Austria) in order to later automatically extract short videos from long videos. The videos were edited using the Ultra-Key functionalities of Adobe Premiere Pro CC (2018) to substitute the green screen backdrop with a grey background and to rectify any color inaccuracies caused by reflections of the green screen on the participants’ faces. Next, a square grid was superimposed on the videos, with the face occupying precisely three-quarters of the height of the square and the nasion aligned in the center. The width of the faces varied accordingly. The videos were then segmented into short clips using FFmpeg (The FFmpeg Developers, 2020), which was embedded in MATLAB (The MathWorks, 2020) for batch-processing, such that each clip contained a single piece of content, such as a sentence or a word. To prevent abrupt starts or endings, each clip commenced 15 frames (0.5 s) before the onset of the content and concluded 15 frames (0.5 s) after the offset of the content. However, there were several instances where participants did not pause for a sufficient duration between two pieces of content, blinked, or moved their gaze away from the camera, resulting in the need to slightly adjust this time frame before or after the content. All clips are in a square format and have been reduced in size to a resolution of 1000 × 1000 pixels. Files are available in .mp4 format. FFmpeg (The FFmpeg Developers, 2020) was used to extract video-only files and audio files from these short audiovisual clips, respectively. Video-only files are available in .mp4 format, audio files are available in .wav format. Static images of neutral facial expressions were retrieved by defining a video frame using Loopy (Loopbio GmbH, Vienna, Austria), in which the actor did not speak, their mouth was closed, their head was not tilted, and facial expression was neutral. These frames were then exported using FFmpeg (The FFmpeg Developers, 2020) and are available in .png format.

### Objective measurements

Several features have been related to attractiveness such as averageness, symmetry, or sexual dimorphism for the face (Little et al., 2011) and averageness (Bruckert et al., 2010) or fundamental frequency for the voice (Mook & Mitchel, 2019). In order to increase the utility of this database for attractiveness research, we quantified a selection of these features according to established methods listed below, using landmarks from the neutral still images for faces and analysis of .wav files for voices. Moreover, the annotated facial landmarks allow further processing with established face manipulation software (e.g., Psychomorph; Tiddeman, 2011).

#### Facial landmarks

**Fig. 3:**
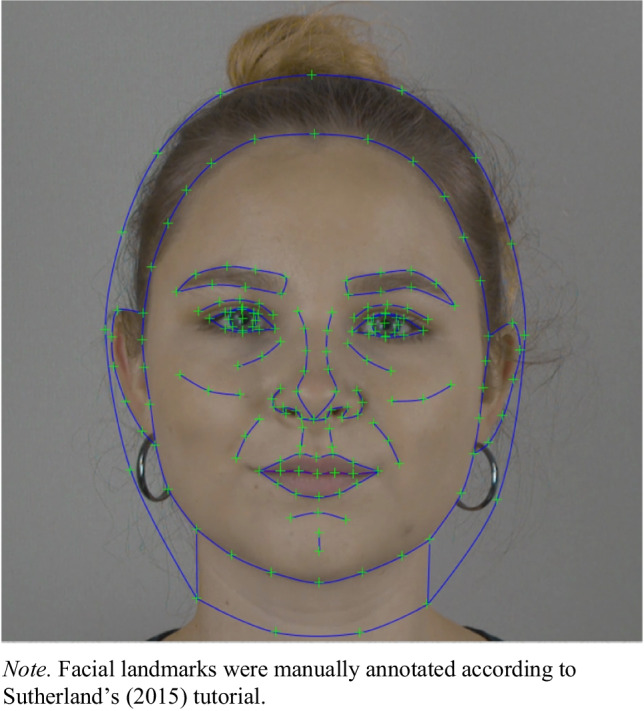
Example face image annotated with 189 facial landmarks according to Sutherland’s guidelines (Sutherland, 2015)

Facial landmarks were positioned on each neutral face image of all 40 participants using Webmorph (DeBruine, 2018), a web-based version of Psychomorph with several additional functions. We placed 189 points on each face, following Sutherland’s guideline (Sutherland, 2015), shown in Fig. 3. The resulting files are provided as .tem files with identical titles to the corresponding face images. They can be uploaded to Webmorph (DeBruine, 2018) for editing or to perform further transformations such as averaging, scrambling, symmetrizing, or morphing. It should be noted that some annotations are merely estimations due to hair-covered portions of the face, with the facial shape and position of the ears not being fully visible.

#### Sexual dimorphism

Following Lee et al. (2014), we measured face-shape sexual dimorphism from the neutral face images using a subset of 132 out of 189 facial landmarks described above. We employed a discriminant analysis method (Lee et al., 2014) and a vector analysis method (Holzleitner et al., 2014). Both methods use generalized Procrustean analysis (GPA) and principal component analysis (PCA) to extract facial shape from landmarks and to calculate either the probability of the face being categorized as male (discriminant analysis) or to locate the face on a continuum from female to male (vector analysis method). In both cases, a higher score indicates a more masculine face shape. The model used to calculate these scores was built within the dataset, i.e., on the faces from the current dataset. Therefore, values of sexual dimorphism are relative to each other and represent masculinity/femininity compared to other stimuli within the dataset. Code for calculating sexual dimorphism is available via https://osf.io/tbkp7 (Holzleitner et al., 2019) and all scores for our sample can be accessed via https://osf.io/hdgmz.

#### Distinctiveness

Following Lee et al. (2016), we measured face-shape distinctiveness using GPA and PCA on facial landmarks. This method measures the distance of the individual face shape from the mathematical average face shape of the sample images. Higher scores represent more distinctive face shapes. Distinctiveness values of facial shape can be inversed to retrieve averageness values of facial shape. Code for calculating distinctiveness is available via https://osf.io/wvxut (Holzleitner et al., 2018) and all scores for our sample can be accessed via https://osf.io/hdgmz.

#### Fundamental frequency

Fundamental frequency is closely related to voice pitch, whereby fundamental frequency refers to the physical phenomenon and voice pitch to our perception of it, i.e., how high or low we perceive a voice. Fundamental frequency was measured for each voice with the spoken content *Morgens ist auf den Straßen viel los* [The streets are busy in the morning]. We used Praat’s (Boersma & Weenink, 2007) autocorrelation function (Boersma, 1993) with input parameters set at 100 Hz for pitch floor, 600 Hz for pitch ceiling, and 0.0075 s as measurement interval. Fundamental frequency ranged from 171.36 to 266.78 Hz in female speakers, and from 104.78 to 157.94 Hz in male speakers. An overview of fundamental frequencies in our sample can be accessed via https://osf.io/hdgmz.

### Subjective ratings

To provide an extensive validation of the database, we also report subjective ratings. We conducted two online validation studies via Labvanced (Finger et al., 2017), one with the purpose of collecting measurements of several dimensions of social perception specifically important for the study of attractiveness and one to validate the different emotional expressions requested of the actors and their recognizability.

#### Ratings of social perception

In this first validation study, we collected ratings of multiple dimensions of social perception, including attractiveness, beauty, and other relevant factors, to provide a comprehensive overview of the various qualities that contribute to social perception. All analyses were conducted using R (version 4.2.2; R Core Team, 2022) and RStudio (version 2022.12.0; Posit team, 2022). For each dimension of social perception, we provide common descriptive statistics. We also report independent *t* tests comparing audiovisual video and static image ratings across all dimensions. Moreover, we will report correlations between all dimensions (aggregated over raters). Throughout, we present *p* values that are considered significant at the level of *α* = 0.05 unless otherwise specified. All analyses are available via https://osf.io/u893v.

##### Raters

A total of 202 raters with a mean age of 24.53 years (*SD* = 6.33 years; 126 female, 74 male, one diverse, one other gender) contributed rating data, of which 175 were psychology students receiving course credit for their participation (Sona Systems, n.d.) and 31 were recruited through the Vienna CogSciHub: Study Participant Platform, which uses the hroot software (Bock et al., 2014), where they received a monetary compensation of 5€. There were no restrictions on sexual orientation as all raters rated all female and male video or image stimuli (*N*_heterosexual_ = 169, *N*_bisexual_ = 22, *N*_homosexual_ = 6, *N*_other sexual orientation_ = 5). Most raters were German native speakers (*n* = 182) or indicated very good (*n* = 17) or good (*n* = 2) German language skills. Raters were randomly assigned to either a *picture group* rating neutral face images or a *video group* rating neutral videos with audio, leading to a slightly different group size with 105 raters assigned to the picture group (64 female, 39 male, one diverse, one other gender; *M* = 23.97 years, *SD* = 4.90 years) and 97 raters assigned to the video group (62 female, 35 male; *M* = 25.13 years, *SD* = 7.57 years).

##### Stimuli and rating scales

As the different emotional conditions were validated in a subsequent study, in this validation study, participants only rated images and videos from the neutral condition. We used 40 neutral still images (of 20 male and 20 female participants) and 40 neutral dynamic videos (also of 20 male and 20 female participants) with the phrase *Hallo, ich bin’s* [Hello, it’s me]. Videos were played with sound. We included dimensions of social perception that have been closely linked to attractiveness in previous studies (e.g., Kuraguchi et al., 2015, investigated attractiveness, sexual attractiveness, and beauty; Little et al., 2011, review distinctiveness, sexual dimorphism, and health). All dimensions were rated on a seven-point Likert scale ranging from 1 “not at all” to 7 “extremely”. We included ratings of general attractiveness (How attractive do you find this person?), of sexual attractiveness (How sexually appealing do you find this person?), and of beauty (How beautiful do you find this person?). Additionally, we measured perceived distinctiveness by assessing memorability to keep the scale comparable and instructions short (How memorable do you find this person compared to others?), sexual dimorphism (How typically female or male do you find this person?), and health (How healthy does the person appear to you?). Moreover, we included personality ratings that have been shown to be related to attractiveness, such as likeability (Zäske et al., 2020), trustworthiness (McGloin & Denes, 2018), and dominance (Bryan et al., 2011). Hence, we asked raters to indicate perceived likeability (How likeable do you find this person?), trustworthiness (How trustworthy does the person appear to you?), and dominance (How dominant does the person appear to you?).

##### Procedure

After reading a welcome message and giving informed consent, participants were randomly assigned to either the picture group or the video group. In the video group, there was an additional headphone task with three trials at the beginning, in which participants were asked to indicate whether they could hear a particular sound. Afterwards, participants of both groups filled out the demographic questionnaire. They received the experimental instructions and continued then with three practice trials, which were designed to familiarize them with the task and, for the video group, to adjust the volume of their headphones if needed. Instructions were similar for both groups: Participants were told to first fixate the fixation cross and that they will be then presented with a picture (or a video) of a person, that they should subsequently rate based on various questions. They were also told that the order of these questions will vary between pictures (videos). After the practice trials, participants were informed that the actual experiment would start. Each participant completed 40 experimental trials. Each trial began with a fixation cross that lasted for 2000 ms, continued with the presentation of an image (with a fixed duration of 5000 ms) or the presentation of a video (duration depending on video length, which varied between 1734 and 3000 ms). Afterwards, a rating page appeared with all nine questions in randomized order, each to be rated on a seven-point Likert scale. Participants did not receive any instructions on how long they should take for the ratings. Finally, after all trials were completed, participants were asked several questions about the experimental procedure to control for confounding variables in online experiments. They indicated whether they knew any of the depicted persons, experienced any technical difficulties or other disturbances, if their way of rating changed over time, and they described their understanding of the experimental task. They could also report other feedback to the researchers.

##### Results

Analyses for this study were mainly descriptive. Our goal was to provide a detailed validation of ratings related to attractiveness, the main research purpose of the database. Moreover, as one of the most valuable characteristics is its multimodality, this validation data was gathered for different stimulus modalities – static images and audiovisual videos. Table 1 summarizes means and standard deviations for each rating dimension, averaged across stimuli, within stimulus gender, and stimulus modality and for all stimuli. Descriptive statistics of each rated stimulus were also calculated and can be accessed via https://osf.io/yu3wb. Moreover, Welch's two-sample *t* tests were used to compare mean ratings given for dynamic audiovisual videos and ratings given for static images, i.e., comparing the average rating given by each participant in the audiovisual video group to the averages given by the static image group participants. Due to the exploratory nature of this validation study, we did not correct for family-wise error. Sexual attractiveness was rated significantly higher, however only slightly, in the image group compared to the video group. None of the other comparisons were significant.

**Table 1:** Means and standard deviations of social perception ratings for neutral audiovisual video stimuli (AV) and neutral static image stimuli (I) and *t* test mean comparison between stimulus modalities (AV and I for all stimuli)

Rating	Female stimuli	Male stimuli	All stimuli	
	*M (SD)*	*M (SD)*	*M (SD)*	*t(df)*
Attractiveness				
AV	3.82 (1.64)	3.14 (1.65)	3.48 (1.68)	– 0.02 (190.24) *p* = .984
I	3.73 (1.61)	3.24 (1.66)	3.48 (1.65)	
Total	3.77 (1.63)	3.19 (1.65)	3.48 (1.66)	
Beauty				
AV	4.27 (1.57)	3.48 (1.60)	3.88 (1.63)	0.68 (183.82) *p* = .500
I	4.09 (1.52)	3.52 (1.57)	3.80 (1.57)	
Total	4.17 (1.55)	3.50 (1.58)	3.84 (1.60)	
Dimorphism				
AV	5.18 (1.24)	5.18 (1.28)	5.18 (1.26)	1.86 (188.34) *p* = .064
I	4.97 (1.37)	5.07 (1.31)	5.02 (1.34)	
Total	5.07 (1.31)	5.13 (1.30)	5.10 (1.30)	
Distinctiveness				
AV	4.32 (1.53)	4.20 (1.51)	4.26 (1.52)	1.00 (198.17) *p* = .319
I	4.26 (1.59)	4.10 (1.51)	4.18 (1.55)	
Total	4.29 (1.56)	4.15 (1.51)	4.22 (1.54)	
Dominance				
AV	3.88 (1.55)	3.70 (1.48)	3.79 (1.52)	– 1.34 (199.65) *p* = .182
I	4.00 (1.61)	3.81 (1.49)	3.91 (1.56)	
Total	3.94 (1.58)	3.76 (1.49)	3.85 (1.54)	
Health				
AV	4.80 (1.50)	4.74 (1.54)	4.77 (1.52)	0.51(192.44) *p* = .612
I	4.72 (1.48)	4.72 (1.52)	4.72 (1.50)	
Total	4.76 (1.49)	4.73 (1.53)	4.74 (1.51)	
Sexual Attractiveness				
AV	2.51 (1.63)	2.12 (1.56)	2.32 (1.61)	-1.99(199.48) *p* = .048*
I	2.73 (1.72)	2.32 (1.63)	2.53 (1.69)	
Total	2.62 (1.68)	2.23 (1.60)	2.43 (1.65)	
Likeability				
AV	4.46 (1.50)	4.29 (1.56)	4.38 (1.53)	– 0.16 (198.96) *p* = .874
I	4.38 (1.48)	4.40 (1.47)	4.39 (1.48)	
Total	4.42 (1.49)	4.34 (1.52)	4.38 (1.50)	
Trustworthiness				
AV	4.63 (1.39)	4.21 (1.52)	4.42 (1.47)	– 0.46 (199.83) *p* = .646
I	4.54 (1.41)	4.38 (1.44)	4.46 (1.43)	
Total	4.58 (1.40)	4.30 (1.48)	4.44 (1.45)	

*Note.* Ratings were given on a 7‐point Likert scale from 1 to 7 and the range of rating data for each dimension and subset of data was always 1 to 7. Stimuli were rated by *n* = 202 participants. Participants were randomly assigned to either the audiovisual or the image condition and rated always both female and male stimuli. Audiovisual videos were rated by *n* = 97 participants, static images were rated by *n* = 105 participants. We report summary statistics for both stimulus gender and for each stimulus modality as well as averaged across all participants and stimulus modalities. *AV* = audiovisual video stimulus, *I* = static image stimulus. Independent-samples *t* tests were calculated to compare mean ratings between stimulus modalities across all stimuli (AV and I). Due to the exploratory nature of these comparisons, no family-wise error correction was applied. Significance level was set to *α* = 0.05. * *p* < .05, ** *p* < .01, *** *p* < .001

**Fig. 4:**
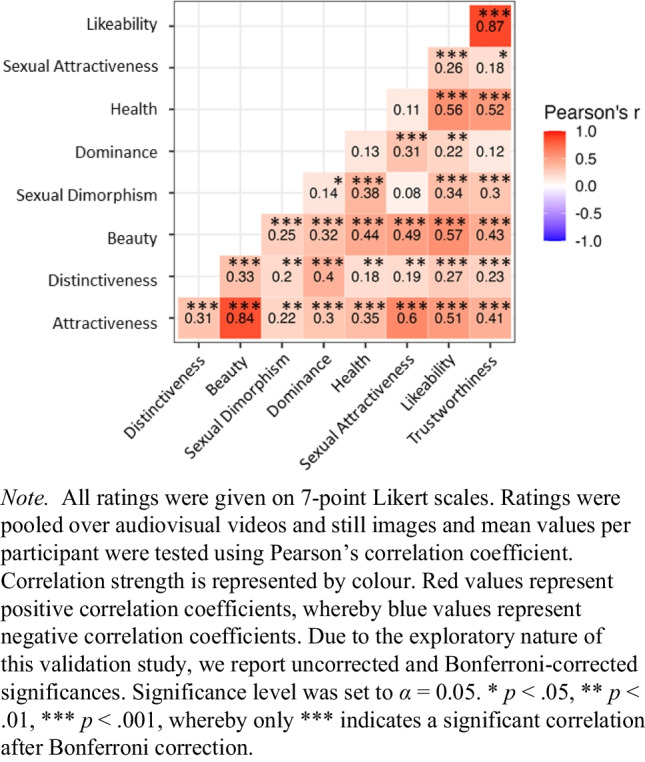
Correlations between rating dimensions of social perception

To examine correlations between each rating dimension (see Fig. 4), Pearson’s correlation coefficient (*r*) was calculated (function *rcorr* in *Hmisc* package; Harrell, 2023) and a two-tailed significance level of α = .05 was used. Audiovisual and image rating were pooled together. We used mean scores per participant for each rating dimension respectively to calculate correlation scores. Due to the exploratory nature of this validation, significant results are reported for both uncorrected and Bonferroni-corrected significance levels (for Bonferroni-correction, significance level was corrected by diving by the number of calculated correlations: α = .05/36 = .001). The strongest positive correlation was found between likeability and trustworthiness, *r* = .87, *p* < .001, followed by attractiveness and beauty, *r* = .84, *p* < .001. All correlations showed at least a trend into the positive direction and most correlations remained significant after Bonferroni correction (indicated by *** in Fig. 4). These results suggest that our judgements of personality traits such as likeability, trustworthiness, or dominance or other underlying qualities such as health are closely linked to external feature ratings such as of attractiveness, beauty, or sexual dimorphism.

#### Emotion validation ratings

In this second validation study, our aim was to evaluate how accurately our audiovisual video stimulus material represents different intended emotional expression categories and how well these emotions are recognized by participants. All analyses are available via https://osf.io/kbuq3.

##### Raters

Fifty-four raters with a mean age of 25.80 years (*SD* = 5.96 years; 44 female, two diverse, eight male) were recruited through the Vienna CogSciHub Study Participant Platform which uses the hroot software (Bock et al., 2014), and received a monetary compensation of 5€. There were no restrictions on sexual orientation as all raters rated all female and male stimuli (*N*_heterosexual_ = 41, *N*_bisexual_ = 7, *N*_homosexual_ = 2, *N*_other sexual orientation_ = 3, N_not specified_ = 1). Most raters were German native speakers (*n* = 53), one rater indicated very good (*n* = 1) German language skills.

##### Stimuli

To validate emotional expressions, we used one of the phrases without deeper meaning *Hallo, ich bin’s* [Hello, it’s me] to offer a variety of prosodic features that are relevant to emotional expression without semantic confounds (Koolagudi & Rao, 2012). Of this spoken content, 195 videos of 36 people with five different expressions (neutral, happy, sad, angry, flirtatious) were available. Three videos are not available in the database (broken files), and two videos were not yet available at the time of the validation.

##### Procedure

**Fig. 5:**
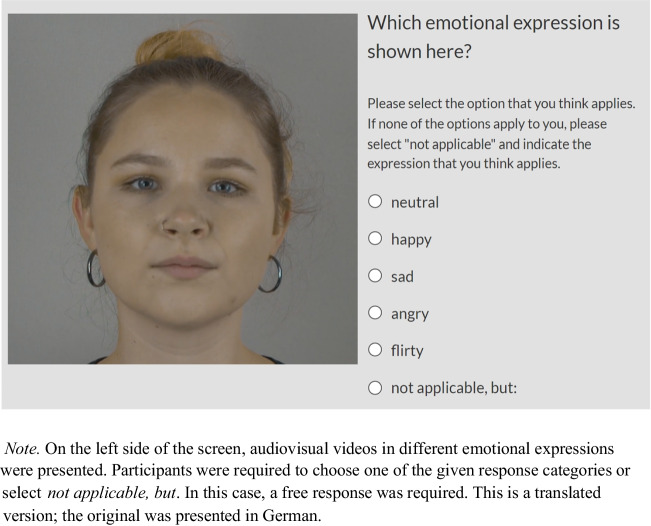
Emotion validation task

Participants were welcomed and gave informed consent. Then, they completed a headphone task with three trials where they were required to indicate if they could hear a specific sound. Next, participants filled out a demographic questionnaire. The experiment began with two practice trials to familiarize participants with the task and adjust headphone volume if necessary. Participants were instructed to watch a 3-s video and select the emotional expression that best matched the person in the video: neutral, happy, sad, angry, flirty, or if they thought none of the expressions applied, they could select “not applicable” and input their own answer. Each participant completed 195 trials, illustrated in Fig. 5, and the duration of each video varied between 1734 and 5248 ms. Answer options were presented simultaneously in a randomized order on the screen, and there was no time limit for participants to complete their ratings. Finally, participants answered questions about the experimental procedure to control for possible confounds, including whether they knew any of the depicted persons, whether they experienced any technical difficulties or other disturbances, whether their way of rating changed over time, and to describe the experimental task. Participants could also provide other feedback to the researchers.

##### Analysis

Emotion category ratings were coded as correct (a value of 1) when the category selected by the rater matched the category of the intended emotion or when the rater chose ‘not applicable’ and named a very similar emotion, e.g., “aggressive” for the category angry, “depressive” for the category sad, or “friendly” for the category happy. These matches were decided post hoc and an overview of our decisions can be accessed via https://osf.io/htcsx. Otherwise, ratings were coded as incorrect (a value of 0). We calculated stimulus-based *proportion correct scores* as measures of accuracy. They represent the proportion of correct responses per category; for *stimulus recognition*, how often emotional expression of the stimulus was correctly categorized divided by the total number of responses that were given by all participants for this stimulus, i.e., each individual file, and for *emotion recognition*, how often an emotion category was correctly identified divided by the total amount of responses from all participants for this emotion category (see also Livingstone & Russo, 2018; Tottenham et al., 2009). With six different answer options, including the option “not applicable”, the chance level of a randomly categorized emotion would be .17, and stimuli or emotion categories, respectively, could be recognized as a distinct emotion when the proportion of correct responses given by all participants surpassed this level. The reader should be aware that this chance level is a pragmatic measure and may not perfectly reflect real-life decisions. The probability of selecting the *not applicable* option is likely lower than choosing any other emotion category, meaning the chance level for selecting a specific target emotion category would be slightly higher. We also calculated actor-based recognition scores, i.e., mean proportion correct scores for each actor, indicating how well each actor’s expressions were recognized, i.e., the number of recognized expressions per actor divided by the number of responses given by all participants for this actor, and how well each actor’s expressions were recognized for each emotion category, i.e., the number of recognized expressions of an emotion category per actor divided by the number of responses given by all participants for this actor in the respective emotion category.

##### Interrater reliability

Fleiss’ kappa was used to assess interrater reliability, i.e., how well raters agree in their response within a certain emotion category (reported in Table 2). Kappa values are interpreted according to Landis' and Koch's (1977) guidelines on the strength of agreement: values < 0 reflect poor agreement, .00 to .20 slight agreement, .21 to .40 fair agreement, .41 to .60 moderate agreement, .61 to .80 substantial agreement, and .81 to 1.00 almost perfect agreement.

**Table 2:** Proportion correct scores and interrater agreement (Fleiss’ kappa) across emotion conditions

Target emotion	Proportion correct scores	Interrater agreement
	*M (SD)*	κ, *p*
Neutral	.74 (.18)	.14, < .001***
Happy	.85 (.17)	.25, < .001***
Sad	.69 (.26)	.32, < .001***
Angry	.78 (.24)	.36, < .001***
Flirtatious	.68 (.26)	.31, < .001***
Overall	.78 (.39)	.30, < .001***

*Note.* Overall scores represent the proportion of correct scores across all target emotion categories and interrater agreement, respectively. Significance level was set to *α* = .005 due to Bonferroni correction to account for multiple comparisons. * *p* < .05, ** *p* < .01, *** *p* < .001

##### Results

Correctness of measures was assessed using stimulus-based proportion correct scores for each emotion category (emotion recognition; Table 2) and for each individual stimulus (stimulus recognition; see https://osf.io/mt5qe). The overall proportion correct score over all emotion conditions was high (*M* = .78, *SD* = .39). Moreover, scores were consistently high across all conditions (all *M*s > .68). Happy and angry expressions scored specifically high (*M* = .85, *SD* = .17 and *M* = .78, *SD* = .24, respectively). Neutral (*M* = .74, *SD* = .18), sad (*M* = .69, *SD* = .26), and flirtatious (*M* = .68, *SD* = .26) scored lower, but can still be considered as high recognition rates among participants. Proportion correct scores for each individual file indicate broad variability between files. We did not instruct participants on the intensity of the intended emotions, resulting in differentially pronounced emotion intensity expressions, which could explain why emotions in some files were easier to detect than in others. Users of the database should take this into account, and moreover, future studies could further validate the database by measuring perceived emotion intensity.

##### Emotion effect on measures of correctness

To assess the effect of emotion on measures of correctness, we calculated a one-way repeated-measures ANOVA with the independent variable *emotion category* with five levels (neutral, happy, angry, and sad) and the dependent variable *proportion correct score*. Three emotion categories (neutral, flirtatious, sad) were non-normally distributed, but visual inspection revealed no serious deviations from normal distribution that would affect a repeated measures ANOVA because it is robust to normality violations (Schmider et al., 2010). Greenhouse–Geisser adjustment of degrees of freedom was applied due to violations of sphericity. We found a main effect for emotion, *F* (2.81, 148.84) = 29.31, *p* < .001,  = .30. Descriptive statistics suggested the following order for recognition rates: Happy (*M* = .85, SD = .17) > Angry (*M* = .78, SD = .24) > Neutral (*M* = .74, SD = .18) > Sad (*M* = .69, SD = .26) > Flirtatious (*M* = .68, SD = .26). These results indicate that happy expressions were recognized correctly most often, while flirting expressions were recognized correctly the least often. Bonferroni-corrected pairwise post hoc comparisons (pairwise *t* tests; reported in Table 3) revealed that most emotion categories significantly differed from each other meaning that emotion category affects recognizability. However, the recognition rates for neutral, sad, and flirting were not significantly different from each other indicating that the ability to recognize these emotions is similar.

**Table 3:** Post hoc comparisons of emotion category recognition

Group 1	Group 2							
	Sad		Neutral		Happy		Flirtatious	
	*T* (53)	*p*	*T* (53)	*p*	*T* (53)	*p*	*T* (53)	*p*
Angry	8.64	< .001**	3.98	< .001**	– 3.92	< .001**	6.33	< .001**
Flirtatious	– 0.04	.972	– 0.99	.326	– 8.23	< .001**		
Happy	9.41	< .001**	7.73	< .001**				
Neutral	0.94	.352						

*Note.* Results of pairwise *t* tests to conduct post hoc comparison to measure effects of emotion category on recognition scores. Group comparisons were always group 1 vs. group 2. Significance level was set to *α* = .005 due to Bonferroni correction to account for multiple comparisons. * *p* < .005, ** *p* < .001

##### Recognition rate per actor

We calculated mean proportion correct scores for each actor, i.e., how well each actor’s expressions were recognized across all emotion categories. Thirty-three out of 40 actors scored higher than .70, 26 actors scored higher than .75, five actors scored over .90. The highest recognition score was .95 and the lowest score was .49, i.e., almost half of the participants correctly rated the intended emotion for the actor with the lowest score. All actors scored over the chance level of recognition of .17. Recognition rates per actor are available via https://osf.io/56azd. Moreover, we provide proportion correct scores for each actor separately for each emotion category. These scores can be accessed via https://osf.io/stjnz.

##### Fleiss’ kappa

Fleiss’ kappa was calculated to assess how raters agree in their emotion categorization (see Table 2). *P* values of Fleiss’ kappa were consistently significant indicating that interrater agreement was significantly different from 0 overall and for each emotion category. Across all target emotion categories, there was fair agreement between raters, κ = .30, *p* < .001. Raters agreed least in categorizing neutral expressions, κ = .14, *p* < .001, and agreed most in categorizing angry expressions, κ = .36, *p* < .001.

## Discussion

In real-life encounters, individuals make social judgements about others based on different sensory modalities. Research has shown that visual and auditory signals are particularly important, for example, in speech recognition that relies on multisensory processing of voices and faces (Campbell, 2007; McGurk & MacDonald, 1976), in emotion perception (Campanella & Belin, 2007), identity processing (Campanella & Belin, 2007), and in attractiveness judgments (Groyecka et al., 2017; Wells et al., 2009). More specifically, there is evidence that visual and auditory information interact with each other (Mook & Mitchel, 2019; Krumpholz et al., 2022) and are integrated, e.g., when judging overall person attractiveness. Therefore, audiovisual stimulus material is crucial to make generalizable statements about social perception. Nonetheless, most available databases only contain stimulus material of one modality, or of both modalities but missing their temporal correspondence. With ViTaFa, we provide a new high-quality audiovisual database allowing research of voice, face, and especially their interaction and integration. ViTaFa is notable for its variety in stimulus modalities (audiovisual dynamic, visual dynamic, visual static, auditory) and diversity of stimuli (including different emotional expressions and a flirting condition) as well as for its extensive subjective and objective validations. The database was created using stimuli from 20 women and 20 men, between 18 and 40 years of age, and is intended for use in studies focusing on human social perception of faces and voices, with a particular emphasis on attractiveness. Additionally, the database is freely available under certain conditions.

In addition to collecting stimuli, our data collection process aimed to provide information to make the database more accessible and valuable to researchers from various fields. Objective measurements were gathered to provide accurate measurements of face and voice and to facilitate their manipulations for research purposes (e.g., face morphing, voice pitch manipulation). We also provide measurements of sexual dimorphism and distinctiveness, which serve as a complement to the subjective social perception ratings we collected to assess the diversity and variability of the database, especially for attractiveness research. This information is of great value specifically for research requiring diverse stimulus material (e.g., a minimum range of attractiveness). We also validated the emotions expressed by the actors in the recorded stimuli. This validation process further enhances the usability of the database in various research applications and extends its applicability in attractiveness research. Future studies can extend the description of the database by providing more subjective data generated under different circumstances or from different populations.

### Availability of the database

The ViTaFa database is currently available for scientific, non-profit research upon request and after signing a confidentiality agreement via https://osf.io/9jtzx. The database comprises pictures of neutral facial expressions (.png format; size, 1000 × 1000 pixels), soundless videos of the faces while pronouncing several different content including letters, words, and sentences (.mp4 format; size, 1000 × 1000 pixels), vocal audio recordings of this content (.wav format), and audiovisual video recordings of this content (.mp4 format, size, 1000 × 1000 pixels). For a precise overview of which files are available for which stimulus person, see https://osf.io/5empg. Moreover, the described validation data of subjective social perception ratings, subjective emotion categorizations, and objective measurements are provided to facilitate and expand the use of the ViTaFa stimuli.

### Limitations and future outlook

The ViTaFa database, while potentially useful for many researchers and research fields, has a few limitations to consider. Firstly, the majority of the sentences in the database are in German, although there are also some pseudo words and letters. This could limit the usefulness of the database for researchers who are interested in studying languages other than German. To keep natural variance, we allowed actors to wear visual ornaments like earrings. While such stimuli are of high ecological validity, it is possible that their presence is an issue for specific research questions. We therefore advise users of the database to keep this in mind when considering ViTaFa for their research. Furthermore, the database has a sample size of 40, with only 20 male and 20 female individuals, which may limit its generalizability to different populations or ages. Nevertheless, there is plenty of various material for each individual. Researchers who are interested in studying various ethnicities or age groups may find the ViTaFa database to be limited in this regard. At present, ViTaFa only contains stimulus material recorded from a frontal perspective. However, uncut recordings from different viewpoints (profile view and ¾ perspective) can be provided on request. This could make it more useful for a broader range of research questions. ViTaFa aims to extend current databases by offering multimodal stimulus material with diverse content and under several emotional conditions. Although ViTaFa can be used across a wide variety of research on social perception, it is also worth mentioning that it is the first database to offer spoken content with flirting expression, a behavior that is often employed to appear more attractive, and therefore ViTaFa offers another possibility specifically for attractiveness research.

## Data Availability

All datasets generated and analyzed in connection to the database and its validation are available via the Open Science Framework registry https://osf.io/9jtzx/. The database itself is available only on request for non-commercial research purposes in order to assure data privacy. Example stimuli are provided. Instructions on how to gain access are described in more detail in the Open Science Framework registry.

## Code availability

Code is available via the Open Science Framework, https://osf.io/9jtzx/.

## References

[CR1] Adobe Systems Incorporated. (2018). *Adobe Premiere Pro CC (Version 12.0)* [Video editing software]. Retrieved May 2020 from https://www.adobe.com/

[CR2] Apostolou M (2021). Involuntary singlehood and its causes: The effects of flirting capacity, mating effort, choosiness and capacity to perceive signals of interest. Personality and Individual Differences.

[CR3] Apostolou M, Papadopoulou I, Christofi M, Vrontis D (2019). Mating performance: Assessing flirting skills, mate signal-detection ability, and shyness effects. Evolutionary Psychology.

[CR4] Audacity Team (2016). *Audacity(R): Free Audio Editor and Recorder (Version 2.1.2)* [Audio editing software]. Retrieved June 2020 from https://audacityteam.org/

[CR5] Back MD, Penke L, Schmukle SC, Sachse K, Borkenau P, Asendorpf JB (2011). Why mate choices are not as reciprocal as we assume: The role of personality, flirting and physical attractiveness. European Journal of Personality.

[CR6] Bahrick LE, Lickliter R (2004). Infants’ perception of rhythm and tempo in unimodal and multimodal stimulation: A developmental test of the intersensory redundancy hypothesis. Cognitive, Affective and Behavioral Neuroscience.

[CR7] Beaupré, M. G., Cheung, N., & Hess, U. (2000). *The montreal set of facial displays of emotion *[Slides]. (Available from Ursula Hess, Department of Psychology, University of Quebec at Montreal, Montreal, Quebec, Canada).

[CR8] Belin P, Fillion-Bilodeau S, Gosselin F (2008). The Montreal Affective Voices: A validated set of nonverbal affect bursts for research on auditory affective processing. Behavior Research Methods.

[CR9] Benda MS, Scherf KS (2020). The Complex Emotion Expression Database: A validated stimulus set of trained actors. PloS One.

[CR10] Bock O, Baetge I, Nicklisch A (2014). hroot – Hamburg registration and organization online tool. European Economic Review.

[CR11] Boersma P (1993). Accurate short-term analysis of the fundamental frequency and the harmonics-to-noise ratio of a sampled sound. IFA Proceedings.

[CR12] Boersma, P., & Weenink, D. (2007). *Praat: doing phonetics by computer (Version 4.5)* [Audio editing software]. Retrieved February 2020 from http://www.praat.org/

[CR13] Bruce V (1994). Stability from variation The case of face recognition the M.D. Vernon Memorial Lecture. The Quarterly Journal of Experimental Psychology.

[CR14] Bruckert L, Bestelmeyer P, Latinus M, Rouger J, Charest I, Rousselet GA, Kawahara H, Belin P (2010). Vocal attractiveness increases by averaging. Current Biology.

[CR15] Bryan AD, Webster GD, Mahaffey AL (2011). The big, the rich, and the powerful: Physical, financial, and social dimensions of dominance in mating and attraction. Personality and Social Psychology Bulletin.

[CR16] Calvo MG, Lundqvist D (2008). Facial expressions of emotion (KDEF): identification under different display-duration conditions. Behavior Research Methods.

[CR17] Calvo MG, Gutiérrez-García A, Beltrán D (2018). Neural time course and brain sources of facial attractiveness vs. trustworthiness judgment. Cognitive, Affective and Behavioral Neuroscience.

[CR18] Campanella S, Belin P (2007). Integrating face and voice in person perception. Trends in Cognitive Sciences.

[CR19] Campbell R (2007). The processing of audio-visual speech: empirical and neural bases. Philosophical Transactions of the Royal Society B: Biological Sciences.

[CR20] Chen JM, Norman JB, Nam Y (2021). Broadening the stimulus set: Introducing the American Multiracial Faces Database. Behavior Research Methods.

[CR21] Chung KM, Kim S, Jung WH, Kim Y (2019). Development and validation of the Yonsei Face Database (YFace DB). Frontiers in Psychology.

[CR22] Cooke M, Barker J, Cunningham S, Shao X (2006). An audio-visual corpus for speech perception and automatic speech recognition. The Journal of the Acoustical Society of America.

[CR23] Cunningham DW, Wallraven C (2009). Dynamic information for the recognition of conversational expressions. Journal of Vision.

[CR24] DeBruine, L., & Jones, B. (2021). *Face research lab london set *[Data set]. Figshare. 10.6084/m9.figshare.5047666.v5

[CR25] DeBruine, L. (2018). *debruine/webmorph: Beta release 2 (Version 0.0.0.9001)* [Web application]. Zenodo. 10.5281/ZENODO.1162670

[CR26] Ebner NC, Riediger M, Lindenberger U (2010). FACES—A database of facial expressions in young, middle-aged, and older women and men: Development and validation. Behavior Research Methods.

[CR27] European Broadcasting Union. (2020). *R 128 - Loudness normalisation and permitted maximum level of audio signals *[PDF]. Retrieved April 25, 2023, from https://tech.ebu.ch/docs/r/r128.pdf

[CR28] Ferdenzi C, Delplanque S, Mehu-Blantar I, Cabral KMDP, Felicio MD, Sander D (2015). The Geneva Faces and Voices (GEFAV) database. Behavior Research Methods.

[CR29] Ferdenzi, C., Ortegón, S. R., Delplanque, S., Baldovini, N., Bensafi, M. (2020). Interdisciplinary challenges for elucidating human olfactory attractiveness. *Philosophical Transactions of the Royal Society B: Biological Sciences*, *375*(1800). 10.1098/rstb.2019.0268 10.1098/rstb.2019.0268 PMC7209927 32306873

[CR30] Finger, H., Goeke, C., Diekamp, D., Standvoß, K., & König, P. (2017). LabVanced: A unified JavaScript framework for online studies. In *Paper presented at the International Conference on Computational Social Science, Cologne*.

[CR31] Fiorentini C, Viviani P (2011). Is there a dynamic advantage for facial expressions?. Journal of Vision.

[CR32] Fujimura T, Suzuki N (2010). Effects of dynamic information in recognising facial expressions on dimensional and categorical judgments. Perception.

[CR33] Gao W, Cao B, Shan S, Chen X, Zhou D, Zhang X, Zhao D (2008). The CAS-PEAL large-scale Chinese face database and baseline evaluations. IEEE Transactions on Systems, Man, and Cybernetics - Part A: Systems and Humans.

[CR34] Garrido MV, Lopes D, Prada M, Rodrigues D, Jerónimo R, Mourão RP (2017). The many faces of a face: Comparing stills and videos of facial expressions in eight dimensions (SAVE database). Behavior Research Methods.

[CR35] Grimm, J., & Grimm, W. (1812/1815). *Kinder- und Haus-Märchen* [Grimm’s fairytales].

[CR36] Gross, R., Matthews, I., Cohn, J., Kanade, T., & Baker, S. (2008). Multi-PIE. In *2008 8th IEEE International Conference on Automatic Face & Gesture Recognition* (pp. 1–8). 10.1109/AFGR.2008.4813399 10.1016/j.imavis.2009.08.002 PMC2873597 20490373

[CR37] Groyecka A, Pisanski K, Sorokowska A, Havlícek J, Karwowski M, Puts D, Craig Roberts S, Sorokowski P (2017). Attractiveness is multimodal: Beauty is also in the nose and ear of the beholder. Frontiers in Psychology.

[CR38] Haq, S., & Jackson, P. J. B. (2010). Multimodal emotion recognition. In W. Wang (Ed.), *Machine Audition: Principles, Algorithms and Systems* (pp. 398–423). 10.4018/978-1-61520-919-4.ch017

[CR39] Harrell Jr, F. E. (2023). *Hmisc: Harrell Miscellaneous (Version 5.0-1)* [R package]. Retrieved from https://CRAN.R-project.org/package=Hmisc

[CR40] Hays J, Wong C, Soto FA (2020). FaReT: A free and open-source toolkit of three-dimensional models and software to study face perception. Behavior Research Methods.

[CR41] Hill AK, Puts DA, Weekes-Shackelford V, Shackelford TK (2016). Vocal attractiveness. Encyclopedia of Evolutionary Psychological Science.

[CR42] Ho PK, Newell FN (2020). Turning heads: The effects of face view and eye gaze direction on the perceived attractiveness of expressive faces. Perception.

[CR43] Holland CAC, Ebner NC, Lin T, Samanez-Larkin GR (2019). Emotion identification across adulthood using the Dynamic FACES database of emotional expressions in younger, middle aged, and older adults. Cognition & Emotion.

[CR44] Holzleitner IJ, Hunter DW, Tiddeman BP, Seck A, Re DE, Perrett DI (2014). Men’s facial masculinity: When (body) size matters. Perception.

[CR45] Holzleitner, I. J., DeBruine, L. M., Lee, A. J., & Jones, B. C. (2018, October 1). *Distinctiveness* [Script]. Retrieved from https://osf.io/wvxut

[CR46] Holzleitner, I. J., DeBruine, L. M., Lee, A. J., & Jones, B. C. (2019, June 24). *Sexual**dimorphism* [Script]. Retrieved from https://osf.io/tbkp7

[CR47] Horstmann G, Ansorge U (2009). Visual search for facial expressions of emotions: A comparison of dynamic and static faces. Emotion.

[CR48] Huang, G. B., Mattar, M., Berg, T., & Learned-Miller, E. (2008). Labeled faces in the wild: A database for studying face recognition in unconstrained environments. Paper presented at the *Workshop on Faces in “Real-Life” Images: Detection, Alignment, and Recognition.* Retrieved from https://inria.hal.science/inria-00321923

[CR49] Janisch J, Mitoyen C, Perinot E, Spezie G, Fusani L, Quigley C (2021). Video recording and analysis of avian movements and behavior: Insights from courtship case studies. Integrative and Comparative Biology.

[CR50] Kaisler, R. E., Marin, M. M., Leder, H. (2020). Effects of emotional expressions, gaze, and head orientation on person perception in social situations. *SAGE Open*, *10*(3). 10.1177/2158244020940705

[CR51] Karras, T., Laine, S., Aila, T. (2018). A style-based generator architecture for generative adversarial networks. *ArXiv*, 4401–4410. 10.1109/tpami.2020.2970919 10.1109/TPAMI.2020.2970919 32012000

[CR52] Koolagudi SG, Rao KS (2012). Emotion recognition from speech: A review. International Journal of Speech Technology.

[CR53] Krumpholz, C., Quigley, C., Ameen, K., Reuter, C., Fusani, L., Leder, H. (2022). The effects of pitch manipulation on male ratings of female speakers and their voices. *Frontiers in Psychology*, *13*(July). 10.3389/fpsyg.2022.911854 10.3389/fpsyg.2022.911854 PMC9302589 35874336

[CR54] Kuraguchi, K., Taniguchi, K., Ashida, H. (2015). The impact of baby schema on perceived attractiveness, beauty, and cuteness in female adults. *SpringerPlus, 4*(1). 10.1186/s40064-015-0940-8 10.1186/s40064-015-0940-8 PMC4393829 25883888

[CR55] Lander K (2008). Relating visual and vocal attractiveness for moving and static faces. Animal Behaviour.

[CR56] Landis JR, Koch GG (1977). The measurement of observer agreement for categorical data. Biometrics.

[CR57] Langner O, Dotsch R, Bijlstra G, Wigboldus DHJ, Hawk ST, van Knippenberg A (2010). Presentation and validation of the Radboud Faces Database. Cognition and Emotion.

[CR58] Lee AJ, Mitchem DG, Wright MJ, Martin NG, Keller MC, Zietsch BP (2014). Genetic factors that increase male facial masculinity decrease facial attractiveness of female relatives. Psychological Science.

[CR59] Lee AJ, Mitchem DG, Wright MJ, Martin NG, Keller MC, Zietsch BP (2016). Facial averageness and genetic quality: Testing heritability, genetic correlation with attractiveness, and the paternal age effect. Evolution and Human Behavior.

[CR60] Lindeberg S, Craig BM, von Lipp O (2019). You look pretty happy: Attractiveness moderates emotion perception. Emotion.

[CR61] Little AC (2014). *Facial attractiveness*. *WIREs*. Cognitive Science.

[CR62] Little AC, Jones BC, Debruine LM (2011). Facial attractiveness: Evolutionary-based research. Philosophical Transactions of the Royal Society B: Biological Sciences.

[CR63] Livingstone, S. R., Russo, F. A. (2018). The Ryerson audio-visual database of emotional speech and song (ravdess): A dynamic, multimodal set of facial and vocal expressions in north American English. *PLoS ONE*, *13*(5). 10.1371/JOURNAL.PONE.0196391 10.1371/journal.pone.0196391 PMC5955500 29768426

[CR64] Lloyd EP, Deska JC, Hugenberg K, McConnell AR, Humphrey BT, Kunstman JW (2019). Miami University deception detection database. Behavior Research Methods.

[CR65] LoBue, V., & Thrasher, C. (2015). The Child Affective Facial Expression (CAFE) set: validity and reliability from untrained adults. *Frontiers in Psychology*, *5*. 10.3389/fpsyg.2014.01532 10.3389/fpsyg.2014.01532 PMC4285011 25610415

[CR66] Longmore CA, Tree JJ (2013). Motion as a cue to face recognition: Evidence from congenital prosopagnosia. Neuropsychologia.

[CR67] Lord KR, Putrevu S, Collins AF (2019). Ethnic influences on attractiveness and trustworthiness perceptions of celebrity endorsers. International Journal of Advertising.

[CR68] Ma DS, Correll J, Wittenbrink B (2015). The Chicago Face Database: A free stimulus set of faces and norming data. Behavior Research Methods.

[CR69] Markham D, Hazan V (2002). The UCL speaker database. Speech, Hearing and Language: UCL Work in Progress.

[CR70] Matsumoto, D., & Ekman, P. (1994). Commentary on “A new series of slides depicting facial expressions of affect” by Mazurski and Bond (1993). *Australian Journal of Psychology, 46*(1), 58. 10.1080/00049539408259471

[CR71] McGloin R, Denes A (2018). Too hot to trust: Examining the relationship between attractiveness, trustworthiness, and desire to date in online dating. New Media and Society.

[CR72] McGurk H, MacDonald J (1976). Hearing lips and seeing voices. Nature.

[CR73] McKone, E., Dawel, A., Robbins, R. A., Shou, Y., Chen, N., Crookes, K. (2021). Why the other-race effect matters: Poor recognition of other-race faces impacts everyday social interactions. *British Journal of Psychology*, 1–23. 10.1111/bjop.12508 10.1111/bjop.12508 34010458

[CR74] Meissner CA, Brigham JC, Butz DA (2005). Memory for own-and other-race faces: A dual-process approach. Applied Cognitive Psychology.

[CR75] Mook AT, Mitchel AD (2019). The role of audiovisual integration in the perception of attractiveness. Evolutionary Behavioral Sciences.

[CR76] Munhall KG, Gribble P, Sacco L, Ward M (1996). Temporal constraints on the McGurk effect. Perception & Psychophysics.

[CR77] Nagrani A, Chung JS, Xie W, Zisserman A (2020). Voxceleb: Large-scale speaker verification in the wild. Computer Speech & Language.

[CR78] Ng H, Winkler S (2014). A data-driven approach to cleaning large face datasets. IEEE International Conference on Image Processing (ICIP).

[CR79] O’Hagen S, Johnson A, Lardi G, Keenan JP (2003). The effect of relationship status on perceived attractiveness. Social Behavior and Personality.

[CR80] Pantic M, Valstar M, Rademaker R, Maat L (2005). Web-based database for facial expression analysis. IEEE International Conference on Multimedia and Expo, ICME.

[CR81] Parsons CE, Young KS, Craske MG, Stein AL, Kringelbach ML (2014). Introducing the oxford vocal (OxVoc) sounds database: A validated set of non-acted affective sounds from human infants, adults, and domestic animals. Frontiers in Psychology.

[CR82] Phillips PJ, Wechsler H, Huang J, Rauss PJ (1998). The FERET database and evaluation procedure for face-recognition algorithms. Image and Vision Computing.

[CR83] Pisanski K, Feinberg DR (2018). Vocal attractiveness. The Oxford Handbook of Voice Perception.

[CR84] Posit team (2022). RStudio: Integrated Development Environment for R. Posit Software.

[CR85] Puts DA, Bailey DH, Cárdenas RA, Burriss RP, Welling LLM, Wheatley JR, Dawood K (2012). Women’s attractiveness changes with estradiol and progesterone across the ovulatory cycle. Hormones and Behavior.

[CR86] R Core Team (2022). R: A language and environment for statistical computing.

[CR87] Raines RS, Hechtman SB, Rosenthal R (1990). Physical attractiveness of face and voice - Effects of positivity, dominance, and sex. Journal of Applied Social Psychology.

[CR88] Reis HT, Wilson IM, Monestere C, Bernstein S, Clark K, Seidl E, Franco M, Gioioso E, Freeman L, Radoane K (1990). What is smiling is beautiful and good. European Journal of Social Psychology.

[CR89] Righi G, Peissig JJ, Tarr MJ (2012). Recognizing disguised faces. Visual Cognition.

[CR90] Rubenstein AJ (2005). Variation in perceived attractiveness: Differences between dynamic and static faces. Psychological Science.

[CR91] Samaria, F. S., & Harter, A. C. (1994). Parameterisation of a stochastic model for human face identification. In *Proceedings of 1994 IEEE Workshop on Applications of Computer Vision* (pp. 138–142). 10.1109/ACV.1994.341300

[CR92] Sanderson, C., & Lovell, B. C. (2009). Multi-region probabilistic histograms for robust and scalable identity inference. In M. Tistarelli & M. S. Nixon (Eds.), *Advances in Biometrics. ICB 2009.* (pp. 243-252). Lecture Notes in Computer Science, vol 5558. Springer. 10.1007/978-3-642-01793-3_21

[CR93] Saribay, S. A., Biten, A. F., Meral, E. O., Aldan, P., Trebicky, V., & Kleisner, K. (2018). The Bogazici face database: Standardized photographs of Turkish faces with supporting materials. *PLoS ONE*, *13*(2). 10.1371/journal.pone.0192018 10.1371/journal.pone.0192018 PMC5812588 29444180

[CR94] Schmider E, Ziegler M, Danay E, Beyer L, Bühner M (2010). Is it really robust? Reinvestigating the robustness of ANOVA against violations of the normal distribution assumption. Methodology.

[CR95] Schmitt DP (2013). Sexual strategies across sexual orientations: How personality traits and culture relate to sociosexuality among gays, lesbians, bisexuals, and heterosexuals. Handbook of the Evolution of Human Sexuality.

[CR96] Sneddon I, McRorie M, McKeown G, Hanratty J (2012). The Belfast induced natural emotion database. IEEE Transactions on Affective Computing.

[CR97] Sona Systems. (n.d.). *Sona Systems: Cloud-based Participant Management Software* [Software]. Sona Systems, Ltd. https://www.sonasystems.com/citation_guide/

[CR98] Strohminger N, Gray K, Chituc V, Heffner J, Schein C, Heagins TB (2016). The MR2: A multi-racial, mega-resolution database of facial stimuli. Behavior Research Methods.

[CR99] Sumby WH, Pollack I (1954). Visual contribution to speech intelligibility in noise. The Journal of the Acoustic Society of America.

[CR100] Sutherland, C. (2015). *A basic guide to Psychomorph*.

[CR101] The FFmpeg Developers. (2020). *FFmpeg (Version 4.3)* [Software]. Retrieved February 2020 from https://www.ffmpeg.org/

[CR102] The Math Works. (2020). *MATLAB**(Version 9.8 (R2020a))* [Software]. Retrieved January 2020 from https://www.mathworks.com/

[CR103] Tiddeman, B. (2011). Facial feature detection with 3D convex local models. In *2011 IEEE International Conference on Automatic Face and Gesture Recognition and Workshops, FG 2011,* (pp. 400–405). 10.1109/FG.2011.5771433

[CR104] Todorov A, Dotsch R, Porter JM, Oosterhof NN, Falvello VB (2013). Validation of data-driven computational models of social perception of faces. Emotion.

[CR105] Tottenham N, Tanaka JW, Leon AC, Mccarry T, Nurse M, Hare TA, Marcus DJ, Westerlund A, Casey BJ, Nelson C (2009). The NimStim set of facial expressions: Judgments from untrained research participants. Psychiatry Research.

[CR106] Ueda, R., Kuraguchi, K., & Ashida, H. (2016). Asymmetric effect of expression intensity on evaluations of facial attractiveness. *SAGE Open*, *6*(4). 10.1177/2158244016677569

[CR107] van der Schalk J, Hawk ST, Fischer AH, Doosje B (2011). Moving faces, looking places: Validation of the Amsterdam Dynamic Facial Expression Set (ADFES). Emotion.

[CR108] Wagner HL (1993). On measuring performance in category judgment studies of nonverbal behavior. Journal of Nonverbal Behavior.

[CR109] Wallhoff F, Schuller BW, Hawellek M, Rigoll G (2006). Efficient Recognition of Authentic Dynamic Facial Expressions on the Feedtum Database.

[CR110] Wehrle T, Kaiser S, Schmidt S, Scherer KR (2000). Studying the dynamics of emotional expression using synthesized facial muscle movements. Journal of Personality and Social Psychology.

[CR111] Wells T, Dunn A, Sergeant M, Davies M (2009). Multiple signals in human mate selection: A review and framework for integrating facial and vocal signals. Journal of Evolutionary Psychology.

[CR112] Zäske R, Skuk VG, Golle J, Schweinberger SR (2020). The Jena Speaker Set (JESS) - A database of voice stimuli from unfamiliar young and old adult speakers. Behavior Research Methods.

